# Azithromycin in the extremely low birth weight infant for the prevention of Bronchopulmonary Dysplasia: a pilot study

**DOI:** 10.1186/1465-9921-8-41

**Published:** 2007-06-05

**Authors:** Hubert O Ballard, Michael I Anstead, Lori A Shook

**Affiliations:** 1 Pediatrics, University of Kentucky, 800 Rose Street, Lexington, KY40536, USA

## Abstract

**Background:**

Azithromycin reduces the severity of illness in patients with inflammatory lung disease such as cystic fibrosis and diffuse panbronchiolitis. Bronchopulmonary dysplasia (BPD) is a pulmonary disorder which causes significant morbidity and mortality in premature infants. BPD is pathologically characterized by inflammation, fibrosis and impaired alveolar development. The purpose of this study was to obtain pilot data on the effectiveness and safety of prophylactic azithromycin in reducing the incidence and severity of BPD in an extremely low birth weight (≤ 1000 grams) population.

**Methods:**

Infants ≤ 1000 g birth weight admitted to the University of Kentucky Neonatal Intensive Care Unit (level III, regional referral center) from 9/1/02-6/30/03 were eligible for this pilot study. The pilot study was double-blinded, randomized, and placebo-controlled. Infants were randomized to treatment or placebo within 12 hours of beginning mechanical ventilation (IMV) and within 72 hours of birth. The treatment group received azithromycin 10 mg/kg/day for 7 days followed by 5 mg/kg/day for the duration of the study. Azithromycin or placebo was continued until the infant no longer required IMV or supplemental oxygen, to a maximum of 6 weeks. Primary endpoints were incidence of BPD as defined by oxygen requirement at 36 weeks gestation, post-natal steroid use, days of IMV, and mortality. Data was analyzed by intention to treat using Chi-square and ANOVA.

**Results:**

A total of 43 extremely premature infants were enrolled in this pilot study. Mean gestational age and birth weight were similar between groups. Mortality, incidence of BPD, days of IMV, and other morbidities were not significantly different between groups. Post-natal steroid use was significantly less in the treatment group [31% (6/19)] vs. placebo group [62% (10/16)] (p = 0.05). Duration of mechanical ventilation was significantly less in treatment survivors, with a median of 13 days (1–47 days) vs. 35 days (1–112 days)(p = 0.02).

**Conclusion:**

Our study suggests that azithromycin prophylaxis in extremely low birth weight infants may effectively reduce post-natal steroid use for infants. Further studies are needed to assess the effects of azithromycin on the incidence of BPD and possible less common side effects, before any recommendations regarding routine clinical use can be made.

## Background

Bronchopulmonary dysplasia (BPD) is a common disorder among extremely low birth weight infants, with an incidence ranging from 30% to 75% in infants weighing less than a 1000 grams at birth[1]. It is a major cause of morbidity and mortality in the preterm infants and has been associated with long-term pulmonary function abnormalities[2], prolonged oxygen therapy, reactive airway disease, and respiratory infections frequently requiring rehospitalization. All of these pose a significant financial burden on the healthcare system[3].

Development of BPD is associated with antenatal and postnatal factors that lead to an arrest of lung development [4-6]. Antenatally, maternal chorioamnionitis is associated with later development of BPD[7], with Ureaplasma urealyticum being the most common association with chorioamnionitis at less than 30 weeks gestational age[8]. The role of Ureaplasma urealyticum antenatally and postnatally is yet to be determined. There is evidence that Ureaplasma urealyticum causes an inflammatory response[9,10], but evidence is conflicting regarding its role in the pathogenesis of BPD [11-14]. Postnatal insults include oxygen toxicity, barotrauma and volutrauma from mechanical ventilation, and sepsis. These additional insults lead to activation of the inflammatory cascade and significantly increase the risk of BPD.

The prevention and treatment of BPD to date has primarily focused on the use of dexamethasone because of its potent anti-inflammatory effect. The use of dexamethasone, however, has not been without significant side-effects. Studies evaluating the efficacy of early postnatal dexamethasone and hydrocortisone were terminated early secondary to increased complications, including high rates of gastrointestinal perforation with both medications[15,16]. Yeh, et al, reported that infants treated postnatally with dexamethasone had significant motor and cognitive impairment when compared with matched controls. Schreiber et al used nitric oxide as an anti-inflammatory agent. They demonstrated a 14% decrease in the incidence of BPD in infants surviving to discharge without evidence of adverse outcome[17]. Although nitric oxide appears to be marginally effective in prevention of BPD, larger clinical trials by Kinsella et al have demonstrated a possible neuroprotective role[18]. Other therapies that have been utilized for the treatment of BPD include vitamin A, which reduces the incidence of BPD by 5–7%[19].

Macrolide antibiotics have anti-inflammatory properties and are thought to inhibit inflammation at multiple points in the inflammatory cascade. Anti-inflammatory actions include inhibiting pro-inflammatory cytokines, inhibiting crucial inflammatory transcription factors, and acting as free radical scavengers. Macrolides are also known to directly inhibit neutrophil chemotaxis and inhibit superoxide generation by activated neutrophils [20-24]. These actions at multiple points in the inflammatory cascade make macrolide antibiotics potentially useful in any chronic inflammatory condition.

Macrolides have been used with success in the treatment of other inflammatory pulmonary diseases, including diffuse panbronchiolitis, cystic fibrosis, and post-transplant bronchiolitis obliterans [25-29]. Macrolides are known to accumulate intracellularly and inhibit neutrophil migration to sites of inflammation[24]. They are also known to inhibit various pro-inflammatory cytokines such as IL-1, IL-6, and TNF-α, as well as NF-κB, a primary transcription factor in the inflammatory cascade[20-24,30]. Newer generation macrolides, such as azithromycin and clarithromycin, are characterized by fewer side-effects and increased anti-inflammatory properties when compared to erythromycin [31-34]. Additionally, azithromycin has increased antimicrobial activity against Ureaplasma urealyticum when compared to erythromycin[35,36].

The inflammatory nature of BPD is clearly established, but a safe and affordable therapy that will decrease its severity and incidence is lacking. Azithromycin, a macrolide antibiotic, has potent anti-inflammatory properties and an excellent side effect profile, but it has not been studied in premature infants. We performed a pilot study to evaluate the efficacy and safety of azithromycin in the prevention of BPD. Our hypothesis is that prophylaxis with azithromycin is both safe and effective at reducing the incidence and severity of BPD in the extremely preterm infant.

## Methods

The pilot study was double blinded, placebo controlled, randomized, and approved by the Institutional Review Board at the University of Kentucky. Infants admitted to the University of Kentucky Neonatal Intensive Care Unit from September 2002 to June 2003 were eligible for enrollment into our study. Enrollment criteria included birth weight ≤ 1000 grams, intermittent mechanical ventilation ≤ 12 hrs duration, and ≤ 72 hours of age. Informed consent was obtained when the infant met enrollment criteria.

Exclusion criteria included lack of parental informed consent, confirmed sepsis by blood culture drawn at admission, multiple congenital anomalies or known syndromes, and intrauterine growth retardation with birth weight less than tenth percentile for gestational age. Infants who did not receive mechanical ventilation were not eligible for the study.

The University of Kentucky Investigational Drug Service performed randomization in blocks of six. Upon enrollment each infant was randomized to receive azithromycin or placebo for a total of 6 weeks. The treatment group received 10 mg/kg of azithromycin daily for 7 days, followed by 5 mg/kg of azithromycin for an additional 5 weeks. This dosing regimen was selected based on prior studies in cystic fibrosis using a 3 month course of azithromycin [26], combined with the nature of BPD being a sustained inflammatory disease. Study drug and placebo were initially administered intravenously, and switched to the enteral route once the infant reached full enteral feeds. The placebo group received an equivalent volume of normal saline daily for 6 weeks. The study drug or placebo was discontinued in the event that infants weaned from mechanical ventilation and supplemental oxygen prior to completion of 6 weeks.

Tracheal aspirates were collected at enrollment for Ureaplasma urealyticum and Mycoplasma culture. Cultures were collected using sterile technique with 0.9% saline lavage. Samples were immediately placed on ice and processed in the clinical laboratory by inoculating REMEL's Micro Test™, a selective transport media formulated for Ureaplasma and Mycoplasma species. Subsequently they were transported frozen to ARUP laboratories in Salt Lake City, Utah for Ureaplasma and Mycoplasma species culture. If an infants respiratory culture was positive for either Ureaplasma or Mycoplasma, the infant was removed from the study secondary to uncertainty regarding the role of these organisms in the pathogenesis of BPD. The selected macrolide and duration of therapy for infants with positive cultures for Ureaplasma or Mycoplasma was at the discretion of the attending neonatologist caring for the infant.

Baseline tracheal fluid aspirates for cytokine determination were collected at enrollment, on day 3 of treatment, and weekly thereafter while intubated and on study. Sterile saline (0.9%) was instilled into the endotracheal tube in four aliquots of 0.5 cc. Fluid was collected into a sterile trap by tracheal suctioning after installation of each aliquot. After the retrieval of the fourth aliquot of saline the catheter was cleared with 0.5 cc of normal saline. Aspirates were transported on ice and processed immediately. The aspirate was spun for 10 minutes at 1000 g and the supernatant frozen at -70°C. Samples were analyzed in triplicate by ELISA for IL-8 (Quantikine^® ^Human IL-8 Immunoassay, Minneapolis, MN) according to the manufacturer's protocol, and the reported value of each sample averaged.

Clinical data were collected at enrollment, on days 3, 5, 7, then weekly for the duration of the study, and at discharge. Data collected included demographic information, mechanical ventilation parameters, blood gas values, mean arterial blood pressure, doses and type of surfactant therapy, total fluids, episodes of bacterial and fungal sepsis, episodes of feeding intolerance (defined as feeds being held for more than 24 hours once initiated), time to full feedings (defined as when the total parental nutrition was discontinued), intraventricular hemorrhage (IVH) diagnosed by head ultrasonography (HUS), periventricular leukomalacia (PVL) seen on HUS, episodes of necrotizing enterocolitis (NEC) Bell's grade II or higher, hearing screen results, hepatic function test (AST), bronchodilator therapy, diuretic therapy, caffeine therapy, and hospital days.

Primary outcomes were incidence of BPD defined as supplemental oxygen at 36 weeks post-menstrual age, duration of mechanical ventilation, post-natal steroid use, and mortality. Post-natal steroids were administered at the discretion of the attending neonatologist.

This pilot study was terminated after 9 months of enrollment to perform data analysis for data safety monitoring, and estimation of sample size. The end-points were the incidence and severity of BPD, with the severity being measured by the duration of mechanical ventilation and post-natal steroid use.

All infants who survived to discharge were scheduled for follow-up at 12 and 24 months corrected age in our NICU graduate clinic for history, physical examination and developmental evaluation. Their neurodevelopmental assessment included Bayley Scales of Infant Development II (BSID-II) and Pre-school Language Scale-4 (PLS-4). Assessments were performed by certified personnel who were blinded to the infants randomization.

Categorical data was analyzed using Chi-square or Fisher's exact test where appropriate, and continuous data was analyzed using ANOVA or median test. JMP^® ^(Cary, NC) software was used for all data analysis.

## Results

**Figure 1:**
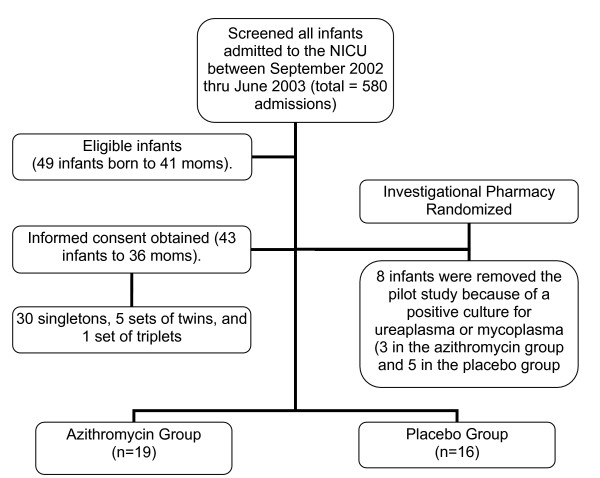
**Flow diagram of patients**. Demonstrates the flow of patients for the pilot study.

From September 2002 to June 2003 there were a total of 49 infants admitted to the University of Kentucky Neonatal Intensive Care Unit who met the eligibility criteria for our study (Figure 1). These infants were born to a total of 41 mothers, of which 36 signed informed consent for their infant to be enrolled in the study. Of the 49 eligible infants, 43 were enrolled (30 singletons, 5 sets of twins, and 1 set of triplets). Reasons for not enrolling the infants included not being able to meet the time criteria (3 cases), the parent not being available for consent (1 case), and the parents refusing participation (1 case). Eight infants had respiratory cultures that were positive for mycoplasma or ureaplasma at the time of enrollment, and were removed from the data analysis. Table 1 compares the study and control groups according to demographic and clinical information at enrollment. Groups were similar as to birth weight, gestational age, gender, cesarean section, and use of antenatal steroids.

**Table 1:** Base-line characteristics for all patients enrolled in the study

Characteristic	Azithromycin Group (n = 19)	Placebo Group (n = 16)	p-value
Birth weight-g	762 ± 94	736 ± 103	NS
Gestational age-wk	25.6 ± 1.5	25.3 ± 1.0	NS
Male sex-no. (%)	7 (36.8)	8 (50)	NS
Mother's racial group-no. (%)			
Black	1 (4.5)	0	NS
White	17 (89.5)	16 (100)	
Other	1 (4.5)	0	
Born at study hospital-no. (%)	17 (89.5)	13 (81.3)	NS
Antenatal Steroids-no. (%)	15 (78.9)	12 (75)	NS
Cesarean section-no. (%)	17 (89.5)	14 (87.5)	NS
Apgar score at 1 minute			
Median	5	4	NS
Interquartile range	2–6	1–7	
Apgar score at 5 minute			
Median	7	7	NS
Interquartile range	6–8	5–8	
Initial mean airway pressure	6.8 ± 1.3	6.6 ± 0.9	NS
Surfactant-no. of doses	1.4 ± 0.7	1.8 ± 0.7	NS
Clinical history of chorioamnionitis-no. (%)	1 (5.2)	1 (6.2)	NS

All data analysis was performed using ANOVA (mean ± sd), except for apgar scores, which was analyzed using median test. All p-values are recorded as NS (not significant) if > 0.05.

The primary outcomes analysis of all respiratory culture negative infants showed equivalent mortality 26.3% (5 of 19) for the azithromycin group as compared to 25% (4 of 16) for the placebo group (p = 0.9). Incidence of BPD was 64.3% (9 of 14) for the azithromycin group vs. 83.3% (10 of 12) for the placebo group (p = 0.26). Median duration of mechanical ventilation was 10 days (range 1–145) for the azithromycin group vs. 16 days (range 1–112) for the placebo group (p = 0.4). Post-natal steroid use to facilitate weaning from mechanical ventilation was significantly less in the azithromycin group with 31.5% (6 of 19) vs. 62.5% (10 of 16) in the placebo group (p = 0.05) receiving steroids (Table 2).

**Table 2:** Outcomes for all patients

	Azithromycin Group (n = 19)	Placebo Group (n = 16)	p-value
Mortality-no. (%)	5 (26.3)	4 (25)	0.9
Incidence of BPD-no. (%)	9 (64.3)	10 (83.3)	0.26
Duration of Mechanical Ventilation-median (range)	10 (1–145)	16 (1–112)	0.40
Postnatal-steroid	6 (31.5)	10 (62.5)	0.05
Days of CPAP (range)	6 ± 7	6 ± 6	0.84
Grade III or IV IVH-no. (%)	5 (26)	5 (33)	0.65
PVL on HUS-no. (%)	1 (5)	3 (19)	0.20
Abnormal Transaminases (AST)	0	1	0.20
Bilirubin Peak	6.5 ± 2	6.3 ± 1.9	0.68
Days to full feeds‡	18 ± 5	18 ± 54	0.95
Feeding intolerance*-no. (%)	5 (26)	9 (56)	0.07
Necrotizing Enterocolitis-no. (%)	0	1 (6)	0.20
Abnormal hearing screen-no. (%)	0	3 (25)	0.08
Bacterial Infection:			
Blood-no. (%)	10 (52)	6 (38)	0.35
Urine-no. (%)	1 (5)	4 (25)	0.15
CSF-no. (%)	0	2 (12)	0.20
Fungal Infection:			
Blood-no. (%)	3 (16)	2 (12)	0.78
Urine-no. (%)	0	2 (12)	0.20
CSF-no. (%)	1 (5)	0	1
Days of Antibiotics§	10 ± 10	19 ± 21	0.10
Caffeine Therapy-no. (%)	13 (68)	10 (62)	0.71
PDA†-no. (%)	10 (52)	12 (75)	0.16
Courses of indomethacin			0.49
Median	1	1	
Interquartile range	0–1	0–2	
PDA ligated-no. (%)	3 (15)	4 (25)	0.49
Diuretics-no. (%)	6 (31)	8 (50)	0.26
Days of Diuretics	14 ± 30	21 ± 38	0.53
Bronchodilators-no. (%)	5 (26)	8 (50)	0.14
Days of Bronchodilators	10 ± 25	18 ± 24	0.34
Hospital days	67 ± 40	78 ± 50	0.47

All data analysis was performed using ANOVA (mean ± sd), except for indomethacin and days of mechanical ventilation, which was analyzed using median test. *Defined as feeds being held for more than 24 hours, while on study. †Confirmed by echocardiogram. ‡Defined as when TPN was discontinued. §Excluding azithromycin.

Secondary outcomes and data safety monitoring showed no difference in the following: IVH Grade III or IV, PVL, abnormal liver function test, peak bilirubin, days to full feedings, feeding intolerance, necrotizing enterocolitis (NEC), abnormal hearing screen, bacterial or fungal infections, antibiotic usage, methylxanthine therapy, incidence of PDA, indocin therapy, PDA ligation, days of CPAP, hospital days, oxygen concentration at 36 weeks PCA, diuretic or bronchodilator use (Table 2).

**Figure 2:**
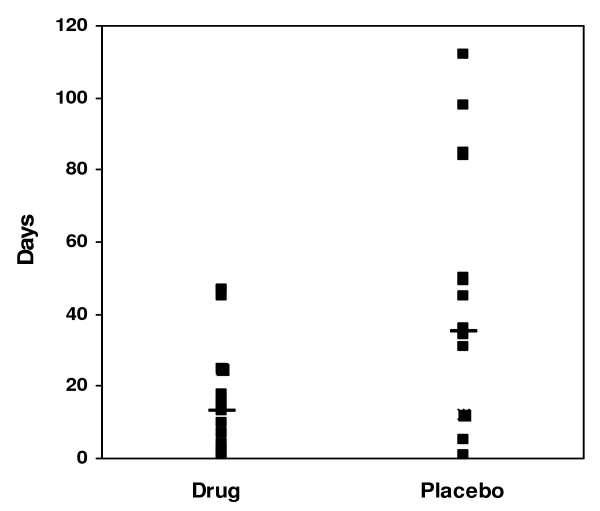
**Days of mechanical ventilation for survivors**. Demonstrates the days of mechanical ventilation for the survivors. The data were analyzed using median test (- represents the median for each group). The p-value is 0.02 between the groups.

Analysis of data from survivors showed a significant reduction in days of mechanical ventilation with a median of 13 days (range 1–47) for the azithromycin group vs. median 35 days (range 1–112) for the placebo group (p = 0.02) (Figure 2). Post-natal steroid use continued to be significantly less in the azithromycin group, with 3 of 16 vs. 8 of 12 in the placebo group receiving steroids (p = 0.01) (Table 3).

**Table 3:** Outcomes for survivors

	Azithromycin Group (n = 14)	Placebo Group (n = 12)	p-value
Incidence of BPD-no. (%)	9 (64.3)	10 (83.3)	0.26
Duration of Mechanical Ventilation-median (range)	13 (1–47)	35 (1–112)	0.02
Postnatal-steroid	3 (18.8)	8 (66.7)	0.02
Days of CPAP (range)	8 ± 7	8 ± 6	0.98
Grade III or IV IVH-no. (%)	2 (14)	2 (16)	0.86
PVL on HUS-no. (%)	1 (7)	2 (16)	0.58
Abnormal Transaminases (AST)	0	1 (8)	0.46
Bilirubin Peak	6.9 ± 1.8	6.4 ± 2.1	0.54
Days to full feeds‡	17 ± 5	18 ± 4	0.76
Feeding intolerance*-no. (%)	4 (29)	7 (58)	0.12
Necrotizing Enterocolitis-no. (%)	0	1 (8)	0.46
Abnormal hearing screen-no. (%)	0	3 (25)	0.08
Bacterial Infection:			
Blood-no. (%)	8 (57)	5 (42)	0.43
Urine-no. (%)	0	3 (25)	0.08
CSF-no. (%)	0	2 (17)	0.20
Fungal Infection:			
Blood-no. (%)	2 (14)	1 (8)	1.0
Urine-no. (%)	0	1 (8)	0.46
CSF-no. (%)	1 (7)	0	1.0
Days of Antibiotics§	12 ± 11	24 ± 23	0.09
Caffeine Therapy-no. (%)	13 (92)	10 (83)	0.58
PDA†-no. (%)	7 (50)	9 (75.0)	0.18
Indomethacin¥			
Median	1	1	0.48
Interquartile range	0–1	0–2	
PDA ligated-no. (%)	3 (21)	3 (25)	0.82
Diuretics-no. (%)	5 (35)	7 (58)	0.24
Days of Diuretics	12 ± 25	28 ± 42	0.23
Bronchodilators-no. (%)	4 (28)	8 (67)	0.05
Days of Bronchodilators	7 ± 15	24 ± 25	0.04
Hospital days	77 ± 15	101 ± 32	0.02

All data analysis was performed using ANOVA (mean ± sd), except for apgar scores and days of mechanical ventilation, which analyzed using median test. *Defined as feeds being held for more than 24 hours, while on study. †Confirmed by echocardiogram. ‡Defined as when TPN was discontinued. §Excluding azithromycin. ¥Performed using wilcoxon rank sum test.

Secondary outcomes for the survivors showed a significant reduction in length of stay (LOS). Treatment group LOS was 77 ± 15 days (range 47–108), vs. 101 ± 32 days (range 49–170), for the placebo group (p = 0.04) (Table 3).

There were a total of 10 infants who did not complete the study. Reasons for not completing the study were death (4 placebo group, 5 azithromycin group), and reversal to a regional hospital (1 azithromycin group). Gestational age and birth weight did not differ between groups. The median days of survival was 4 (range 1–166) for the azithromycin group vs 8 (range 1–21) for the placebo group. Causes of death included hypoxic respiratory failure (2 placebo group, 1 azithromycin group), hyperkalemia (2 azithromycin group), fungal sepsis (1 placebo group, 1 azithromycin group), suspected sepsis (1 placebo group), and cardiopulmonary arrest (1 placebo group). There were no side-effects that could be attributed to the azithromycin therapy during or after our study. Mean arterial pressure, feeding intolerance, hepatic function, peak bilirubin, episodes of apnea receiving treatment with caffeine, episodes of bacterial or fungal sepsis, incidence of PDA, episodes of NEC, incidence of IVH or PVL, and abnormal hearing screens were comparable between the study and placebo groups.

**Figure 3:**
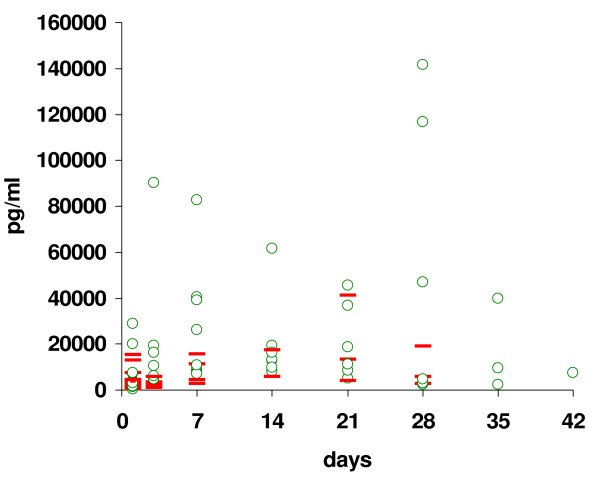
**Tracheal aspirate IL-8 values**. Demonstrates the tracheal aspirate values that were collected at enrollment into the study (day 0) and then study days 3, 7, 14, 21, 28, 35 and 42. The ○ represents values for the control group, and the **- **represents values for the azithromycin group. There are numerous missing values due infants being extubated, and therefore not being able to collect tracheal aspirates. The p-value is not significant between groups.

Results of the tracheal aspirate analysis for IL-8 are shown in figure 3. There are numerous missing values secondary to infants not completing the study due to mortality. Additionally, infants who received azithromycin required mechanical ventilation for a median of 13 days, and therefore did not have tracheal aspirates collected after extubation. The IL-8 results are not statistically different between groups.

Among the survivors, 32 were scheduled for developmental follow-up at 12 and 24 months corrected age, with 22 of 32 (69%) returning for follow-up. The mean corrected age at follow-up was 16.5 ± 5.2 months for azithromycin group and 18.3 ± 5.4 months for the placebo group. There were no differences in the number of hospital re-admissions or days on oxygen after discharge between groups, and no infants had been diagnosed with hypertrophic pyloric stenosis. The results of their growth parameters, BSID-II, and PLS-4 are shown in Table 4.

**Table 4:** NICU graduate clinic follow-up data at 20–24 months on all patients

Parameter	Azithromycin Group (n = 12)	Placebo Group (n = 10)	p-value
Weight (kg) (range)	9.3 (7.5–11.2)	9.9 (6.7–14.2)	0.4
Length (cm) (range)	75.9 (65–84.7)	78.1 (70–86)	0.31
Head Circumference (cm) (range)	45.9 (43–47.8)	46.3 (42–50)	0.69
Bayley (corrected):			
MDI (range)	89 (68–108)	80 (49–105)	0.1
PDI (range)	84 (55–108)	74 (49–100)	0.09
PLS-4 (standard score):			
Auditory Comprehension	87 (74–109)	82 (62–100)	0.31
Expressive Communication	90 (65–108)	76 (62–108)	0.03
Total Language Score	87 (65–108)	77 (62–105)	0.08

Data were analyzed using ANOVA, with mean and ranges. Abbreviations: MDI (Mental Developmental Index), PDI (Psychomotor Developmental Index) and PLS-4 (Pre-school Language Scale-4).

## Discussion

BPD is known to be an inflammatory disease of the lungs that predominantly affects preterm infants[37,38]. Risk factors include prematurity, maternal chorioamnionitis, mechanical ventilation, oxygen therapy, and sepsis[6,39]. Antenatal and postnatal factors contribute to an activation of the inflammatory cascade and subsequent development of BPD. Tracheal aspirates from infants with BPD have increased neutrophil counts as well as elevated pro-inflammatory cytokines including TNF-α, IL-1, and IL-6 [40-43]. The elevated neutrophil counts are thought to contribute to impaired lung morphogenesis by transporting and releasing harmful proteases[44]. These many factors lead to an arrest of lung development which is the hallmark of BPD[5].

Therapies to prevent and treat BPD have remained elusive. Early post-natal steroid use is effective, but not without significant side-effects[15]. Nitric oxide may be beneficial as an anti-inflammatory agent, but at significant cost[17]. Vitamin A therapy is the only treatment that has been demonstrated to be beneficial, with a 5–7% decrease in the incidence of BPD[19]. This study presents pilot data that strongly supports the need for additional evaluation of azithromycin in the treatment and prevention of BPD.

The first macrolide to be used clinically for its anti-inflammatory properties was erythromycin, which improved survival in diffuse panbronchiolitis from less than 50% to about 90%[25]. In patients with cystic fibrosis azithromycin has been shown to improve the forced expiratory volume in 1 second (FEV_1_), decrease respiratory exacerbations requiring hospitalization, and improve quality of life[26]. A recent pilot study showed some possible benefit of azithromycin in bronchiolitis obliterans syndrome by improving FEV_1 _and quality of life in pulmonary transplant patients[28]. Knowing that all of the previously mentioned lung diseases have predominant inflammatory components, it is reasonable to evaluate the role of macrolides in the prevention and treatment BPD.

We designed a pilot study to investigate the anti-inflammatory effects of azithromycin in extremely preterm infants at high risk for BPD. Our pilot study excluded infants who had respiratory cultures that were positive for Ureaplasma urealyticum or Mycoplasma hominis. This was done because of the undetermined role of these organisms in the pathogenesis of BPD [11-14]. There is evidence that Ureaplasma causes an inflammatory response[9,10], but prior studies evaluating treatment of Ureaplasma in preterm infants have not demonstrated any benefit[45,46]. The uncertainty in this area is further demonstrated by the recent Food and Drug Administration request for proposals titled "Effectiveness of intravenous azithromycin for the prevention of bronchopulmonary dysplasia in preterm neonates colonized with Ureaplasma urealyticum".

This study had inadequate sample size to make any conclusions regarding its affect on the incidence of BPD and could not assess for possible side effects that have an exceedingly low incidence. This study was intended to obtain pilot data for a larger trial. Potential concerns for the use of azithromycin include late onset infections, particularly fungal sepsis, antimicrobial resistance, hepatotoxicity, hearing impairment, and other gastrointestinal side-effects especially with a prolonged course of therapy. Possible association with cardiac arrhythmias has not been identified, but would also need to be monitored. We found that the incidence of these were similar to placebo. Although the macrolide erythromycin has been reported to be associated with hypertrophic pyloric stenosis[47], this complication was not seen in our study and has not been reported with azithromycin.

In this pilot study we found no statistically significant decrease in BPD, although the incidence in the azithromycin-treated infants was 19% lower than in the placebo-treated infants. Several measures of the severity of lung disease were significantly decreased in the treated infants, including days on mechanical ventilation and length of stay. These data justify further study of the effects of azithromycin on BPD in the extremely low birth weight infant. Larger trials would need to monitor closely for toxicities associated with azithromycin, and continue to include long-term neurodevelopmental follow-up.

## Conclusion

Our pilot study showed that infants treated with azithromycin prophylaxis were less likely to receive postnatal steroids. Survivors also had shortened duration of mechanical ventilation, and shortened hospital stay when receiving azithromycin prophylaxis. Side effects of azithromycin prophylaxis were minimal and similar to placebo. Further studies are needed before azithromycin can be recommended for routine therapy in the treatment of BPD.
